# Arbuscular mycorrhizal phenotyping: the dos and don'ts

**DOI:** 10.1111/nph.15489

**Published:** 2018-10-13

**Authors:** Hector Montero, Jeongmin Choi, Uta Paszkowski

**Affiliations:** ^1^ Department of Plant Sciences University of Cambridge Cambridge CB2 3EA UK

**Keywords:** arbuscular mycorrhiza (AM), arbuscule, colonization, phenotyping, symbiosis

Most plant lineages engage with Glomeromycotina fungi to form the ubiquitous arbuscular mycorrhizal (AM) symbiosis. Despite its wide occurrence in diverse plant–fungal species combinations, the interaction dynamics are strikingly uniform (Fig. 1). The events leading up to a successful mutualism start when plant and fungus advertise their presence in the rhizosphere by releasing diffusible chemical cues. In vascular plants this presymbiotic dialog results in physical contact whereby extraradical hyphae differentiate into hyphopodia on the surface of roots preceding fungal entry. Intraradical hyphal passage is followed by fungal accommodation in cortical cells to foster arbuscules. This is accompanied by the rapid formation of plant and fungal membranes in juxtaposition to each other, resulting in a magnified surface area. The mutualistic nature of the association manifests here as the reciprocal exchange of nutrients occurs. Subsequently formed fungal vesicles and spores are symptomatic of a sustained association. At the whole‐root level, symbiosis establishment is considered asynchronous as all AM fungal symbiotic structures can be found simultaneously. However, at the infection unit resolution, every stage depends on the preceding one reflecting that precise molecular programs dynamically coordinate the interaction.

**Figure 1:**
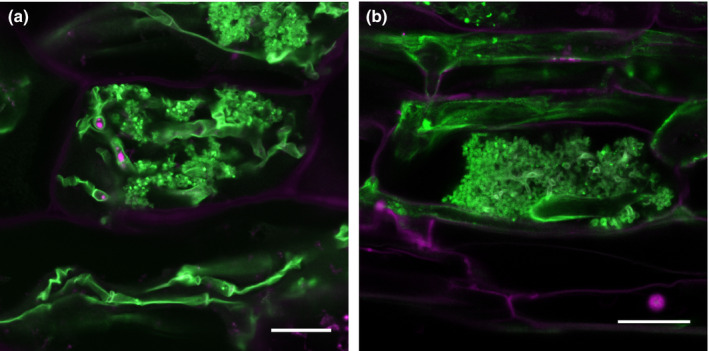
Arbuscular mycorrhizal (AM) fungal symbiotic structures are similarly devised in distantly related plant clades. (a) *Marchantia palacea* thalloid cell and (b) maize cortical root cell colonized with the AM fungus *Rhizophagus irregularis* highlighting the fundamental conservation of symbiotic traits across land plants. WGA‐Alexa Fluor 488 staining. Bars, 20 μm.

Although the knowledge of Glomeromycotina genomics has recently improved (Kamel *et al*., 2017), plants remain experimentally more tractable than the fungal partner leading to a more advanced understanding of the plant regulatory mechanisms. The discovery of functionally relevant genes relies on the accurate characterization of symbiotic phenotypes which often implies documenting the growth of the fungal partner resulting from alterations of plant host genes. This can be a complex task and mutant phenotypes are routinely reported in the absence of standardized procedures leading to ambiguous interpretations. Here we provide general guidelines for accurately characterizing AM phenotypes in their temporal and spatial aspects.

## Importance of systemic diagnoses

AM phenotypes observed during the colonization process can be explained by gene impairment in the plant directly affecting symbiosis establishment and/or functioning, or secondarily as a side effect of abnormal plant development. The possibilities are ample. Alterations in processes related to photosynthesis, cell wall properties, root development and hormonal regulation could all potentially lead to strong symbiotic phenotypes, but the underlying disrupted plant genes causing them do not necessarily need to be dedicated to symbiosis. To distinguish pleiotropic from specific symbiotic phenotypes, plant developmental, fungal and overall symbiotic phenotypes must be comprehensively assessed. Additionally, any conclusion drawn should be contemplated based on the model organisms used. Although any plant pecies able to host AM fungi is a good model to study fundamental symbiotic processes, extrapolations should be avoided when dealing with genes involved in plant clade‐specific dynamics. For example, symbiotic LysM‐containing receptors have undergone expansion in legumes likely followed by neo‐ or sub‐functionalization adapted to the bipartite symbiotic context of this plant family (Gough *et al*., 2018).

## Phenotyping a snowball

**Figure 2:**
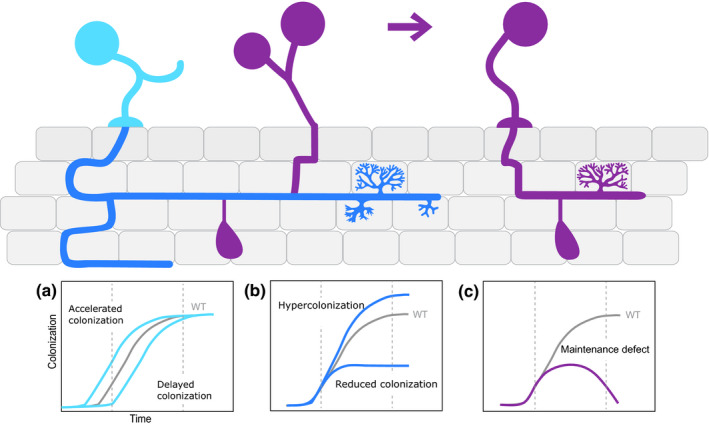
Time course observations can capture the dynamic patterns of arbuscular mycorrhizal (AM) fungal colonization. (a) Success of the presymbiotic stage could affect the time of onset of AM fungal colonization. Altered AM fungal spore germination, hyphal branching and hyphopodia formation could result in accelerated or delayed AM fungal colonization, which is evident at early time points. (b) Intraradical hyphal progression including arbuscule development influences the quantity of the colonization at later time points. (c) Success of post‐arbuscule development stage includes vesicle formation and sporulation. Failure in these processes could affect maintenance of symbiosis resulting in AM fungal colonization collapse at later time points. WT, wild‐type.

The encounter between the root of a young seedling and extraradical fungal hyphae results in a lifelong association in which both parallel and consecutive recolonization events are the norm. Although inherently iterative, it is currently unknown how the first colonization event compares to recolonization at the molecular and physiological level. Priming may render a fraction of the presymbiotic colonization events not needed during recolonization. By contrast, plant cell autonomous events are likely to require the same *de novo*‐activated set of molecular drivers in non colonized and recolonized roots. Although AM fungal colonization of a plant root is a stepwise process, the timing of the different events is plastic and dependent on environmental factors besides plant and fungal identity. Hence alterations in one step of the cyclic procedure of AM fungal colonization will necessarily affect subsequent steps, likely in a dynamic and amplified fashion (Fig. 2). From the phenotyping perspective this complexity demands a proper temporal resolution of AM fungal colonization. The inclusion of multiple time points in experimental designs should be a standard procedure when describing AM fungal colonization, aiming to capture general quantitative dynamics in the presymbiotic and symbiotic stages of the association followed by observations on the development of particular AM fungal symbiotic structures. Several techniques have been developed to stain AM fungi (Vierheilig *et al*., 2005) which are normally used in conjunction with standardized methods to quantify the extent of AM colonization in root tissues (Giovannetti & Mosse, 1980).

To complement microscopic observations, transcript levels of plant and AM fungal marker genes known to be induced at different stages of symbiotic establishment should be monitored. While several AM fungal housekeeping genes are routinely used as a way to indirectly assess fungal biomass, it would be useful to develop a set of AM fungal marker genes induced at distinct symbiotic stages. From the host plant side, several time‐course transcriptomics studies have identified genes related to early and late stages of the association (reviewed in Choi *et al*., 2018; Pimprikar & Gutjahr, 2018). However, the fact that mutation in plant symbiotic genes can potentially affect the expression of marker genes highlights their use as a complement rather than an alternative to microscopic observations.

## Assessing phenotypes in the presymbiotic stage

In the presymbiotic stage, plant roots and AM fungi secrete diffusible compounds in the rhizosphere allowing reciprocal recognition before physical contact. The plant host increases the secretion of signaling molecules, such as strigolactones (SLs; Akiyama *et al*., 2005) from root exudates, in order to metabolically activate AM fungi. Highly ramified fungal mycelia then produce carbohydrate based signaling molecules such as LipoChitoOligosaccharides (LCOs) and ChitoOligosaccharides (COs) (Maillet *et al*., 2011; Genre *et al*., 2013). Successful presymbiotic reprogramming is the prerequisite for subsequent initiation of root colonization as demonstrated by the impairment of initiation of invasion of the roots of plant mutants defective in this process (Gutjahr *et al*., 2015; Nadal *et al*., 2017).

One of the key steps monitoring the initial root colonization by AM fungi is to quantitatively and qualitatively assess hyphopodia formation defects, which are often observed in plant mutants defective in AM fungal perception. For example, mutants of the rice chitin receptor, Chitin Elicitor Receptor Kinase 1 (CERK1) and mutants of genes involved in the common symbiosis pathway showed similar aberrant swollen hyphopodia (Saito *et al*., 2007; Gutjahr *et al*., 2008; Kosuta *et al*., 2011; Liao *et al*., 2012; Miyata *et al*., 2014). Once defects in AM fungal hyphopodia formation are identified, subsequent AM fungal colonization assays with increasing inoculum strength are useful to test whether the phenotype can be overcome thereby demonstrating the robustness of the early defects. Conversely, some plant mutants such as those overproducing SLs (Yoshida *et al*., 2012; Gutjahr *et al*., 2015), exhibit higher AM fungal colonization compared to wild‐type plants. The use of high AM fungal inoculum strength for AM colonization assays can potentially mask these phenotypes, hence assaying increased number of replicates with reduced inoculum is advised.

Strong early phenotypes could be due to the plant's inability to either produce active compounds such as SLs to prime the fungi or to recognize fungal compounds such as LCOs or COs. To distinguish between these two scenarios, global transcriptomics of plant or AM fungi have been implemented to compare transcriptional responses to germinated spore exudates (GSEs) of the AM fungi or plant root exudates, respectively (Campos‐Soriano *et al*., 2011; Giovannetti *et al*., 2015; Gutjahr *et al*., 2015; Nadal *et al*., 2017). Alternatively, nurse plant experiments where mutants are co‐cultivated with wild‐type plants can be performed. If the presymbiosis defects are complemented in the mutant plant, its phenotype likely relates to the production of root exudates rather than being defective in perception of AM fungi. If this is the case, the next step would be to investigate the consequences on AM fungal performance, which includes spore germination, hyphal branching and directional growth towards the root. To this end, *in vitro* AM fungal spore germination or hyphal branching assays with root exudates collected under low phosphate conditions can be performed (Kretzschmar *et al*., 2012; Aroca *et al*., 2013; Nadal *et al*., 2017).

## Phenotyping the arbusculated cell

The development of arbuscules is mirrored by developmental reprogramming of the hosting root cells. The reciprocal exchange of nutrients in arbusculated cells renders them a specialized cell type, which is also evidenced by their specific transcriptional activity as determined in laser‐captured microdissection studies (reviewed in Pimprikar & Gutjahr, 2018). Only a small fraction of the plant genes induced in arbusculated cells has been functionally characterized, mostly in nutrient exchange and peri‐arbuscular membrane (PAM) development (Luginbuehl & Oldroyd, 2017). Impairment in symbiotic processes from the arbusculated cell perspective can potentially fall into vast spectra of outcomes ranging from the extreme cases of complete absence of arbuscules to fully formed ones but with impaired function (Breuillin‐Sessoms *et al*., 2015). Decreased arbuscule numbers could be due to failures in presymbiotic processes, in which case this should be mirrored by decreased abundance of all symbiotic fungal structures. By contrast, the phenotype likely relates to the symbiotic stage if fungal entry and spread in the roots occurs but arbuscules do not form. Hence when reporting a reduced number of arbuscules it is more informative to also document hyphopodia formation and intraradical hyphal spread.

Although the interaction between plant roots and AM fungi is long‐lasting, at the cell level the commitment for symbiosis is ephemeral as arbuscules disappear in a matter of days after their inception. Arbuscule development phenotypes are, in contrast to alterations in arbuscule abundance, less likely to be linked to presymbiotic influences. Rather, they can arise from impairment of genes impacting the accommodation of initial intracellular hyphae, arbuscule size, branching or lifespan. The majority of genes that have been demonstrated to regulate arbuscule developmental processes are of plant origin but some fungal genes have been shown to participate as well (Helber *et al*., 2011; Tsuzuki *et al*., 2016; Xie *et al*., 2016; Sun *et al*., 2018). As wild‐type plants will display arbuscules at diverse developmental stages at a given time point, it is important to score the relative proportions. Quantification of the size of arbuscules in relation to that of the host cell is advised here, which is commonly performed by employing wheat germ agglutinin (WGA) fluorophore‐conjugated treated roots. However, there is a continuum in the transition from a nascent, fully branched to a degenerating arbuscule that makes the interpretation of the developmental stage in place challenging to determine. To examine arbuscule branching, efforts have focused on the implementation of live cell imaging, particularly using subcellular marker lines (Ivanov & Harrison, 2014) including those for the differential localization to PAM subdomains (Park *et al*., 2015). Impairments of a number of plant genes involved in lipid (Keymer & Gutjahr, 2018) and phosphate dynamics (Javot *et al*., 2007; Yang *et al*., 2012) exhibit stunted arbuscules linking nutrient homeostasis with arbuscule development. Using nurse plant systems to investigate complementation of phenotypes provides glimpses on the involvement of the underlying genes in AM fungal nutrition, hence it is advised to perform this experiment routinely.

To track arbuscule collapse, plant subcellular marker lines can be used in combination with time lapse imaging. Such markers have not been broadly implemented mainly because the study of arbuscule collapse is in its infancy. Of potential use are plant peroxisome markers as they have been shown to localize around collapsing arbuscules (Pumplin & Harrison, 2009) and the plant secretory carrier membrane protein SCAMP which forms distinctive aggregates as arbuscules collapse (Kobae & Fujiwara, 2014). The general occurrence of the patterns, however, is yet to be confirmed.

## It is not over when the arbuscule is over

When an arbuscule vanishes from a root cell, new ones are born elsewhere. In parallel, fungal vesicles and spores appear. Vesicles are globular intercellular structures believed to be storage organs for lipids including phospholipids (Jabaji‐Hare *et al*., 1984). As phospholipids potentially contain phosphate and lipids traded between symbionts, alterations in vesicle abundance may reflect major defects in symbiotic nutrient homeostasis. Unlike arbuscules, vesicle formation is stimulated by phosphate treatment in rice (Kobae *et al*., 2016) suggesting them to have complex yet‐to‐be understood roles in the fungal handling of nutritional resources. Vesicles and spores are overlooked structures but they give account of the completion of fungal life cycle which is, in turn, relevant for subsequent recolonization. Post‐arbuscule defects are expected to ultimately lead to the collapse of the association which may be observable only upon prolonged symbiotic co existence. Importantly, it is at this stage that the plant and fungus are concomitantly assimilating the nutrients exchanged, conceivably leading to further modifications of plant physiological processes. This adds a layer of complexity to the phenotyping process that researchers should be aware of as more molecular players appear in this largely unexplored stage of the AM symbiosis.

## Concluding remarks

**Figure 3:**
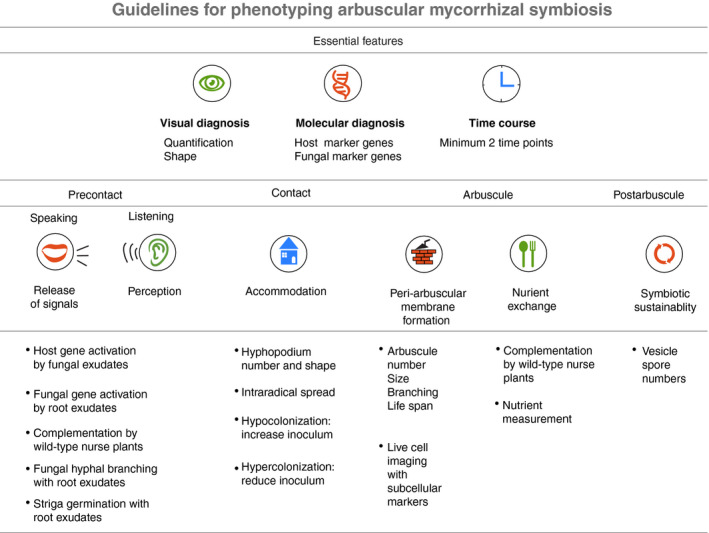
Guidelines for phenotyping arbuscular mycorrhizal (AM) symbiosis. AM phenotyping data should contain three essential features. Visual diagnosis includes AM fungal staining to quantify the colonization level and assess shapes of AM fungal structures. Stage‐specific host and fungal molecular marker gene expression should support the visual observations. Root samples should be harvested at least at two‐time points to capture the early and late stages of AM fungal colonization. This initial evaluation will reveal a specific stage when a plant host gene is likely to function. We listed assays that may help to address roles of host genes in each stage of AM fungal colonization.

Although the researcher's biological question is the ultimate driver of the method of choice for AM symbiosis phenotyping, minimum standards must be followed (Fig. 3). To achieve this, the plant systemic context should be considered alongside phenotypic assessment over plant roots colonized by AM fungi. Upon zooming into the roots, the dynamic cumulative complexity of the AM fungal colonization process cannot be captured in a snapshot. Instead, phenotyping should be performed at multiple time points to distinguish the stage during which symbiotic alterations are occurring. This, together with inspections of alterations in particular AM fungal symbiotic structures, will help unveil functional aspects of the plant genes impaired in an accurate and reproducible way.
